# From radioimmunoassay to mass spectrometry: a new method to quantify orexin-A (hypocretin-1) in cerebrospinal fluid

**DOI:** 10.1038/srep25162

**Published:** 2016-05-11

**Authors:** Christophe Hirtz, Jérôme Vialaret, Audrey Gabelle, Nora Nowak, Yves Dauvilliers, Sylvain Lehmann

**Affiliations:** 1 CHU Montpellier, Institut de Recherche en Biothérapie, hôpital St Eloi, Laboratoire de Biochimie Protéomique Clinique et CRB, Montpellier, F-34000 France; 2 Université de Montpellier, Montpellier, F-34000 France. INSERM U1183, Montpellier, F-34000 France; 3 Memory Research Resources center, Department of Neurology, Gui-de-Chauliac Hospital, Montpellier University Hospital, F-34000 France; 4 National Reference Centre for Orphan Diseases, Narcolepsy, Idiopathic hypersomnia and Kleine-Levin Syndrome, France; 5 Sleep Disorders Center, Department of Neurology, Gui-de-Chauliac Hospital, CHU Montpellier, Inserm U1061, Montpellier, France

## Abstract

I^125^ radioimmunoassay (RIA) is currently the standard technique for quantifying cerebrospinal fluid (CSF) orexin-A/hypocretin-1, a biomarker used to diagnose narcolepsy type 1. However, orexin-A RIA is liable to undergo cross-reactions with matrix constituents generating interference, high variability between batches, low precision and accuracy, and requires special radioactivity precautions. Here we developed the first quantitative mass spectrometry assay of orexin-A based on a multiple reaction monitoring (MRM) approach. This method was tested in keeping with the Clinical and Laboratory Standards Institute (CLSI) guidelines and its clinical relevance was confirmed by comparing patients with narcolepsy type 1 *versus* patients with other neurological conditions. The results obtained using MRM and RIA methods were highly correlated, and Bland–Altman analysis established their interchangeability. However, the MRM values had a wider distribution and were 2.5 time lower than the RIA findings. In conclusion, this method of assay provides a useful alternative to RIA to quantify orexin-A, and may well replace it not only in narcolepsy type 1, but also in the increasing number of pathologies in which the quantification of this analyte is relevant.

The hypocretins (Hcrt), which are also known as orexins, consist of two neuropeptides, orexin-A/hypocretin-1 (Orex-A/Hcrt-1) and orexin-B/hypocretin-2 (Orex-B/Hcrt-2). They originated from the same precursor gene (preprohypocretin) present in a few thousand neurons localized in the perifornical area of the lateral hypothalamus^1^,^2^. The longer peptide of the two, Orex-A/Hcrt-1, contains 33 amino acids, and the shorter one, Orex-B/Hcrt-2, 28 amino acids^3^. After being released, these peptides bind to two 7-transmembrane G-coupled receptors, Hcrt receptor 1 (Hcrt-1R) and 2 (Hcrt-2R)^3^. Hcrt-1R is abundantly expressed in the locus coeruleus and the dorsal raphe nucleus, and has a preferential binding affinity for hypocretin-1, whereas Hcrt-2R is expressed in several brain regions and binds to both forms of Hcrt with a similar affinity^4^,^5^,^6^. Hcrt-containing neurons project widely in the brain to target sites involved in sleep/wake regulation and arousal processes, reward-motivated behavior, endocrine homeostasis, and stress states^7^,^8^,^9^.

More than a decade ago, a dramatic decrease (85–95%) in the number of Hcrt neurons was identified as the cause of human narcolepsy with cataplexy^10^,^11^. Narcolepsy was further divided in the third revised International Classification of Sleep Disorders into narcolepsy type 1 (NT1), which is also known as hypocretin deficiency syndrome, and narcolepsy type 2 (narcolepsy with normal hcrt levels) rather than being classified as previously as narcolepsy with and without cataplexy^12^. Highly specific and sensitive methods of measuring patients’ CSF hcrt-1 levels are therefore required for diagnosing NT1^1^. Recent studies have also suggested that these levels are of interest in patients with neurodegenerative disorders: a relationship is in fact thought to exist, between hcrt-1 and brain accumulation of β-amyloid peptides in Alzheimer’s disease^13^,^14^,^15^.

Orex-A/Hcrt-1 can be measured in crude CSF using the I^125^ Radio-Immuno-Assay (RIA) kit available from Phoenix Pharmaceuticals (Belmont, CA): values below 110 pg/ml (using the Stanford reference sample) are highly specific to NT1^1^. If RIA is often used and currently considered as the standard clinical method to measure CSF Orexin-A^1^,^16^, enzyme immunoassay (EIA)^17^ and fluorescence immunoassay (FIA)^18^ approaches have been also developed to quantify this analyte.

Since it involves the use of radioactive materials, RIA requires specific precautions for the use and disposal of these materials. RIAs have many other disadvantages, including possible cross reactivity with matrix constituents, great variability between batches, and low precision and accuracy. To avoid these constraints, new methods of quantification are therefore required for measuring CSF orexin levels. Approaches such as targeted mass spectrometry (MS) and especially multiple reaction monitoring (MRM) using internal standards can be used to develop quantitative assays with a high level of specificity, precision and robustness^19^. The latest Selected Reaction Monitoring/Multiple Reaction Monitoring (SRM/MRM) methods which have emerged during the last few years for quantifying biomarkers in clinical contexts^19^ are based on the principle of monitoring specific ions called proteotypic peptides and fragments of these ions known as transition fragments. This data-dependent MS approach, which lends itself particularly well to studies in the field of clinical chemistry proteomics^20^, often relies on the use of a triple quadrupole mass spectrometer. Recent multi-site trials confirmed that this approach is compatible with clinical applications in terms of reproducibility and robustness^21^,^22^. If MS appears an interesting alternative to RIA to measure CSF orexin-A levels, this approach has however some disadvantages including the cost of equipment, the complexity of the workflows and the need for high skilled operators.

The innovative method to quantify Orex-A/Hcrt-1 in human CSF described in the present study is based on the use of liquid chromatography combined with mass spectrometry (LC-MS) in the MRM mode with internal standards to ensure absolute quantification. The performance of the method was tested in terms of specificity, reproducibility, and repeatability, as well as its limit of blank (LOB), of detection (LOD), of quantification (LOQ) and the linearity. A clinical validation was then performed comparing LC-MRM and RIA in a selected population of patients with either normal or hypocretin-deficient conditions.

## Results

### Quantification of CSF orexin-A using the LC-MRM method

**Figure 1:**
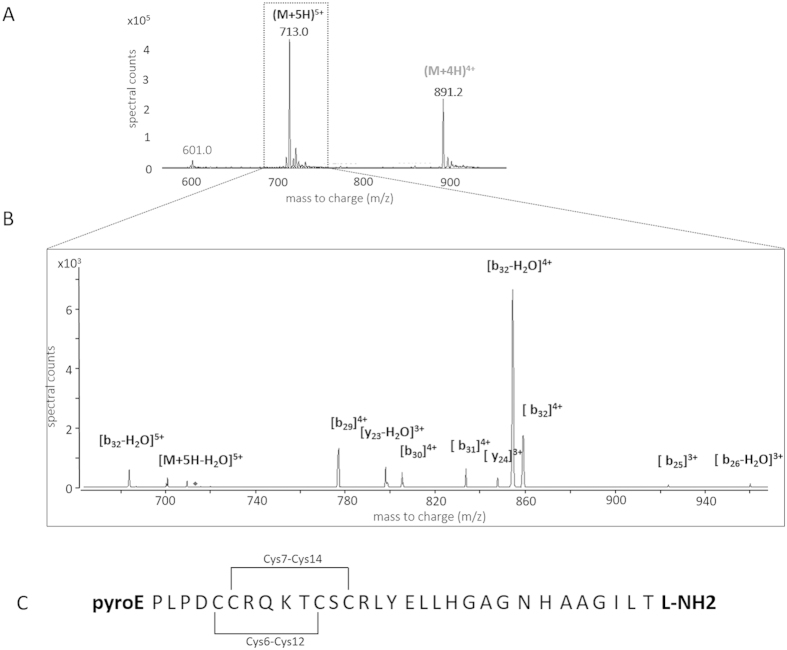
Panel (**A**) MS/MS spectra of the [M+5H]^5+^ precursor of light orexin-A standard. Panel (**B**) Light orexin-A standard chromatograms obtained by micro-HPLC-MS in the “full-scan mode” showing 4+ and 5+ charged molecular ions. Panel (**C**) Orexin-A sequence with modifications (N-ter Pyroglutamination, C-ter amidation, disulfite bridges).

A full scan mass spectrum of the light orexin-A peptide standard was first recorded in order to select parent ions for the LC-MRM measurements. The ions generating the most intense peaks, [M+4H]^4+^ (m/z 891) and [M+5H]^5+^ (m/z 713), which were selected as precursor ions (Fig. 1A,B), were fragmented in a Product Ion Scan (PIS) and the fragments generated were examined. Four transitions per peptide were monitored and one transition (713.3– > 854.1) was used as a quantifier. Based on these results, the MRM method was optimized for the Collision Energy (using Agilent Technologies’ “peptide optimizer” software program). The Collision Energy was optimized for each transition in order to obtain the most sensitive method (Table 1).

The best conditions of sample re-suspension were obtained with 20% acetonitrile/1% formic acid/79% water, storing the samples in a LC on a deactivated glass vial insert. Samples were injected in 15% of the B phase (90% ACN, 0.1% FA) at a flow rate of 0.1 mL/min (Table 2). The total duration of the LC-MRM run was 6 minutes which is compatible with high throughput clinical analyses (the elution of orexin-A was observed after 3.5 min).

### Analytical validation of the LC-MRM orexin-A assay

The analytical precision of the calibration standards (as determined by performing reproducibility tests) ranged from 3 to 14% (Table 3). In human CSF samples with normal orexin-A concentrations (>200 pg/mL on RIA), the analytical precision tested by repeating five times measurement on pooled CSF samples with different values (32.9, 29.6, 39.3, 28.1 and 34.8 pg/mL) was lower than 7%. The mean CV of eleven individual CSF samples with normal CSF orexin-A levels analyzed in duplicate was lower than 15%. The results of the reproducibility tests performed on three different days using calibration standards, ranged from 8 to 21%. A new calibration curve was then drawn up on each series of biological samples in order to reduce the level of analytical variation. The final accuracy values obtained with pooled blank CSF spiked with various amounts of orexin-A standard (14, 25, 50 pg/mL) ranged between 94 and 104% (Table 4).

In order to determine the selectivity of the LC-MRM method, MRM chromatograms of three blank CSF samples were compared with that obtained on a blank CSF sample spiked with 50 pg/mL of light orexin-A standard. The fact that no MRM signal was detected in the chromatograms of the blank CSF samples at the orexin-A retention time confirmed the high selectivity of the method.

### Assessment of the quantification

**Figure 2:**
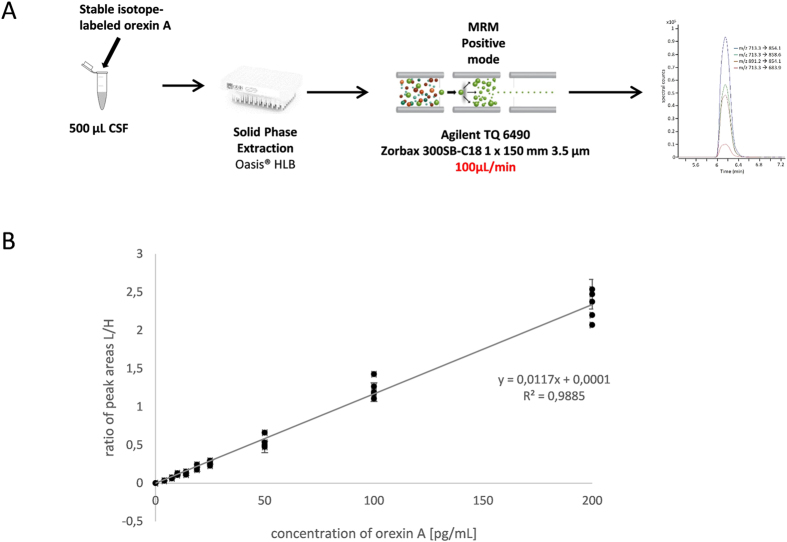
Panel (**A**) Workflow used for the micro-LC-MRM quantification of orexin-A in human CSF samples. Panel (**B**) Calibration curves of orexin-A in the 0–200 pg/mL concentration range (0, 4, 7.5, 10, 14, 19, 25, 50, 100, 200 pg/mL). The equation was linear and r^2^ was equal to 0.9885, based on the following equation: y = 0.0117x + 0.0001.

The calibration curves were generated by performing linear regression analysis and plotting the peak area ratios (light orexin-A standard/labeled orexin) versus the concentration ratios (Table 3). Based on the values obtained with RIA, calibration curves were generated to cover the 0–200 pg/mL concentration range using 10 calibration points (n = 5). Each concentration was tested to detect the presence of outliers (Dixon test, P = 99%) and to determine the normal distribution (David test, P = 99%): the results showed the presence of no outliers. The linearity of the method was confirmed, based on the Pearson correlation criterion r^2^ > 0.99 (Fig. 2B). The LOB was 0.006 pg/mL, the LOD 0.009 pg/mL in H_2_O/ACN/FA (80/20/0.1, v/v/v) and the LOQ was 0.025 pg/mL, based on 3 replicates and 5 measurements. The absence of memory/carry-over effects was confirmed by re-analyzing the calibrator/calibration points without adding SIS at the end of the analytical sequence. The results of LC-MRM quantification were not affected by the dilution of the samples, since the linear regression coefficients consistently showed a high level of linearity (R^2^ > 0.99). In addition, the fact that the inter-day coefficient of variation was lower than 20% on the 4–20 pg/mL range in the case of all the calibration points shows that the orexin-A standard was highly stable at a temperature of 4 °C.

### Matrix effects

Comparison of the MS peak areas of the SIS spiked either in blank CSF or in H2O/ACN/FA 80/20/0.1, v/v/v revealed a matrix related to ion suppression of 97% of the signal intensity. This difference was mainly due to the previously described high molecular complexity of the CSF matrix in comparison with that of the solvent, which impacted the process of ionization^23^. This matrix effects could however be balanced by adding in the CSF isotope-labelled internal standard and by relying on ratios for quantification.

### Comparison between LC-MRM and RIA methods

**Figure 3:**
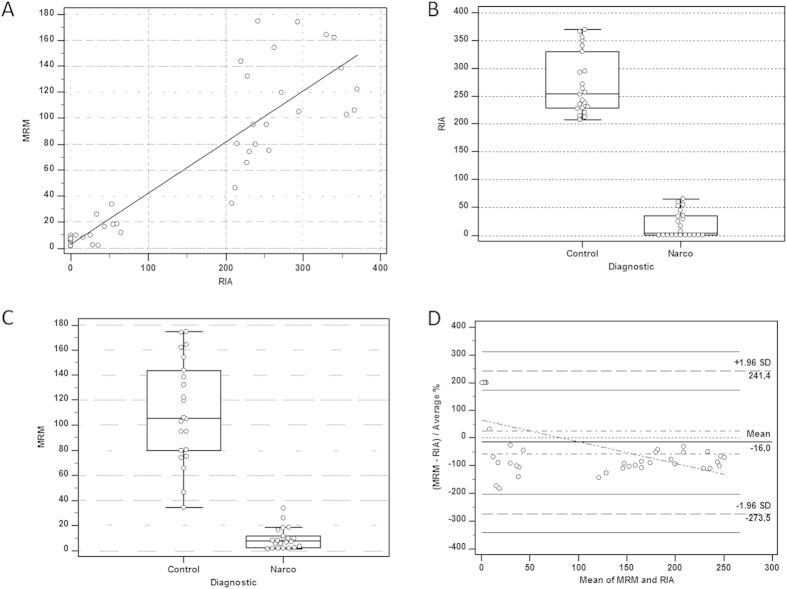
CSF samples from patients with narcolepsy (NT1, n = 22) and various neurological disorders (n = 22) were analyzed using RIA and MRM. Panel (**A**) values plotted show the existence of a significant correlation (Spearman’s rank correlation coefficient (rho) 0.898, significance level P < 0.0001) between the two methods. A clear-cut difference between the absolute values was observed, however, the RIA values were 2.5 times higher than the MRM values. Graph of the RIA Panel (**B**) and MRM values Panel (**C**) obtained in the two clinical groups presented as medians and interquartile ranges. Statistical pairwise comparisons performed with the non-parametric Mann-Whitney test confirmed the difference observed between the two groups of patients (P < 0.000001). Bland–Altman analysis with Deming regression Panel (**D**) confirmed that the two methods were interchangeable since the values of the differences between them were in the +/−1.96 SD range.

CSF samples with normal orexin-A levels (i.e. >200 pg/ml, n = 22 patients with various neurological disorders) and low orexin-A levels (i.e. <110 pg/ml, n = 22 patients with NT1) determined using the standard RIA method were reanalyzed using the LC-MRM method. A significant between-group differences in the CSF orexin-A levels were observed with the two methods (Mann-Whitney test, p < 0.000001) (Fig. 3B,C). To minimize the differences between the RIA and MRM measurements, the RIA standards were used to generate the MRM calibration curve; however, the CSF orexin-A levels obtained using the latter method were 2.5 times lower on average than the RIA values, namely 105 and 254 pg/mL, respectively, in the control group. The orexin-A values were also more widely distributed in the case of the MRM method. We found a significant positive correlation between MRM and RIA results (r2 = 0.898, Spearman’s r P < 0.001) and the Bland-Altman plot (Fig. 3D) validated the commutability of these methods (since the differences between the results obtained were in the +/−1.96 SD range). However, the slope of the linear correlation (Fig. 3A) (0.394x) confirmed that our MRM assay values were more than two times lower than those obtained with RIA. This result is likely related to the fact that MS methods are fully specific (i.e. only orexin-A is quantified) while commercial antibodies might cross react with other molecules. The LC-MRM intra assay CV was 8% as compared to 7% for RIA while the MRM inter-assay CV was 15% as compared to 31% for the RIA. This high inter-assay CV for the RIA method is likely in relation with the variability in antibody batch quality, purity of the peptide and specific activity (counts per minute per one mole of hypocretin) of the labeled I125-hypocretin ligand.

## Discussion

The reliable innovative quantitative approach developed and validated here for measuring CSF Orexin-A/Hcrt-1 using LC-MRM methods was found to show high specificity, reproducibility, repeatability, linearity and low limits of detection and quantification. This new method could well differentiate two populations of patients with normal and low CSF orexin-A (i.e. patients with NT1) but it generated orexin-A values that were more widely distributed.

The demand for means of measuring CSF orexin-A levels in the context of sleep disorders has been increasing since the recent publication of the revised International Classification of Sleep Disorders, in which narcolepsy has been divided into type 1 and 2 depending on CSF hypocretin-1 levels^24^ . Recent studies have also suggested that this parameter may be of particular interest in patients with neurodegenerative disorders and that a relationship may exist between the orexin-A/hypocretin-1 levels and β-amyloid peptides metabolism^13^,^14^,^25^,^26^,^27^.

I^125^ RIA is the standard clinical method currently used to measure orexin-A in CSF because this method is sufficiently sensitive to detect this analyte, which is present in humans in very low amounts. However, RIA has some major drawbacks including poor inter-batch intermediate precision, and the need to take highly stringent and costly precautions for the use and disposal of radioactive material. In addition, this method based on immunocompetition is highly susceptible to interference and liable to generate cross-reactions with metabolized fragments, circulating receptors and other analogous endogenous proteins^28^,^29^. False-positive or negative results may therefore occur and the concentrations recorded may not reflect the actual endogenous values due to a lack of specificity and the variability of the origins and purity of the standards used to generate calibration curves^30^.

One interesting alternative to immunodetection methods is represented by quantitative mass spectrometry in MRM mode that improves assay specificity. This bioanalytical approach can be used to unambiguously identify the target with a high level of reproducibility and robustness^20^, and is currently held to be the most suitable method in the field of clinical chemistry proteomics^31^ as for small molecules and xenobiotics. Importantly, the use of an internal standard helps to reduce variability linked to the sample preparation and to interaction with proteins within the sample^32^. In order to measure the low levels of CSF orexin-A present in humans, we had to develop a pre-fractionation protocol based on the use of Solid Phase Extraction. This method results in the generic depletion of abundant analytes making it possible to perform in-depth proteome analysis^23^. The outstanding analytical performances of our novel LC-MRM method in terms of specificity, reproducibility, repeatability and linearity and limits of detection and quantification make it eminently suitable for clinical applications. If MS appears therefore to be an interesting alternative to RIA, this approach has however some disadvantage including the cost of equipment, the complexity of the workflows and the need for high skilled operators. Comparisons with the existing commercial RIA method showed that the values obtained using the two approaches were highly correlated and could distinguish equally patients with NT1 and other neurological patients. However, a 2.5-fold difference was observed between the CSF orexin-A levels obtained using the LC-MRM and RIA methods, which may have been attributable to immunoassay interference.

As all the patients with NT1 previously tested have been found to show low CSF orexin-A levels (<110 pg/ml) due to the loss of approximately 90% of the orexin-expressing neurons, the highly sensitive standard RIA method has been widely used as a diagnostic tool for discriminating between patients with NT1 and other conditions^10^,^11^,^33^. In a previous study on rats, a 50% decrease in the CSF orexin-A levels was observed when 73% of the orexinergic neurons were lost, but there were some discrepancies between measurements^34^. More than 80% of all patients with narcolepsy with atypical cataplexy, without cataplexy and those with other sleep or neurological disorders were found to have normal CSF orexin-A levels (>200 pg/ml) based on the RIA findings, which suggests that the majority of these patients’ orexinergic neurons remain intact^1^,^16^,^35^. A postmortem study on a patient with narcolepsy without cataplexy recently indicated that a loss of 33% of the orexin-positive cells had occurred, probably due to a partial loss of neurons, which the RIA per se failed to bring to light^36^. Likewise, some discrepancies were previously reported between the total number of orexin-A immunoreactive neurons quantified in postmortem hypothalami (corresponding to a 40% decrease in the number of cells) and the orexin-A concentrations measured in postmortem ventricular cerebrospinal fluid (corresponding to a 14% decrease) from patients with Alzheimer’s disease^37^. RIA has been increasingly used to quantify CSF orexin-A despite its technical drawbacks, with limited and controversial usefulness in clinical contexts so far, however, with the exception of NT1 patients.

In conclusion, the reliable innovative method based on LC-MRM developed and tested here for quantifying CSF orexin-A may therefore well replace RIA in the future. Between-method comparisons on larger populations, including patients with NT2, with other central hypersomnias, with intermediate CSF orexin-A levels based on the RIA findings are needed to replicate and extend our preliminary findings. Further studies are also required to define pathological cutoffs using this alternative method, and to explore the role of orexin-A in the neurobiology of sleep in neurodegenerative disorders.

## Methods

### Characteristics of subjects

In order to test the MRM method, a pool of CSF samples from patients (n = 20) with various neurological disorders but not NT1 was used (RIA concentration of orexin A>200 pg/mL). CSF was collected in polypropylene tubes under standardized conditions. Each CSF sample was sent to the laboratory within 4 hours of being collected and centrifuged at 1000 g for 10 minutes at 4 °C. CSF was aliquoted into 1.5-mL polypropylene tubes and stored at −80 °C until further analysis. To test the validity of the MRM method the following two clinical population samples were set up: 1) 22 drug-free patients (8 females, mean age: 27.7 ± 18.7 and 14 men, mean age: 24.5 ± 12.7) with typical NT1 defined by the presence of sleepiness, clear-cut cataplexy, human leukocyte antigen (HLA) DQB1*0602 positivity, at least two sleep onset REM periods and a mean sleep latency below 8 min during the multiple sleep latency test. All these patients underwent a lumbar puncture prior to the study and were found to have low CSF Orex-A/Hcrt-1 levels (<110 pg/ml) as determined by performing direct I^125^ RIA; and 2) 22 drug-free patients (16 females, mean age: 38.1 ± 16.4 and 6 men, mean age: 42.8 ± 13.9) with various neurological disorders but not NT1. These patients underwent a lumbar puncture prior to the study and were found to have CSF Hcrt-1 levels >200 pg/ml as determined by performing direct I^125^ RIA. Demographic, clinical and neurophysiological parameters of these patients are presented in Table 5.

### Preparation of orexin-A standard solutions for LC-MRM assays

Human orexin-A standard (pyroEPLPDCCRQKTCSCRLYELLHGAGNHAAGILTL-NH_2_) was purchased from Phoenix Scientific and internal orexin standard (pyroEPL*PDCCRQKTCSCRL*YEL*L*HGAGNHAAGIL*T*L-NH_2_) [13C6, 15N] Leu* with a purity >97% as assessed by performing RP-HPLC and MS was purchased from Eurogentec (Seraing, Belgium). Standard peptides were synthesized in lyophilized form with the same amino acid sequence (33 AA), folding pattern (2 disulfide bridges Cys 6-12; 7-14), N-terminal pyroglutamination and C-terminal amidation as the RIA standard (Fig. 1C). Purchased stable isotope standard (SIS) and unlabeled orexin A peptide standards were dissolved in water (water ULC-MS, ref. 23214102)/acetonitrile (acetonitrile ULC-MS (ACN) ref.01204101)/formic acid (formic Acid ULC-MS (FA) ref. 069141A8) (66.2/33.8/0.1, v/v/v) at a concentration of 200 μg/mL. The solution was then separated into aliquots of 50 μL in LoBind tubes (Protein LoBind tube 1.5 mL, ref. 022431081 Eppendorf, Le Pecq, France) and stored at −80 °C. In the case of the MRM method, the standard solutions were then diluted with H_2_O/ACN/FA (80/20/0.1, v/v/v). Appropriate dilutions of 200 μg/mL orexin-A stock solutions were made to prepare the calibration curve in the concentration range of 0–200 pg/mL (0, 4.0, 7.5, 10.0, 14.0, 19.0, 25.0, 50.0, 100.0, 200.0 pg/mL). Samples were spiked with the internal standard at a final concentration of 50 pg/mL.

### CSF sample preparation for LC-MRM assays

500 μL of CSF (corresponding on average to 350 μg of proteins) were acidified with orthophosphoric acid (ref. 452289-50ML, Sigma-Aldrich) (final concentration 1.33%). Simultaneously, the Oasis® SPE HLB μElution plate (ref. 186001828BA) was preconditioned with 350 μL of methanol (MeOH ULC-MS ref. 13684101 all from Biosolve, Dieuze, France), followed by 350 μL of water. Liquids were handled with a vacuum (Waters manifold for 96-well plates). Acidified samples were loaded onto the plate and washed successively with 200 μL of water, 200 μL methanol/water/ammonium hydroxide (NH_4_OH ref. 338818–100ML) (30/65/5 v/v/v), and 200 μL of water. Bound proteins were eluted in two steps with 50 μL methanol/acidified water with 0.1% TFA (90/10 v/v). The eluted samples were dried on a vacuum concentrator (Labconco, Kansas city, USA) and stored at −80 °C. Prior to LC-MRM analysis, the samples were resuspended with 20 μL of H2O/ACN/FA (80:20:0.1 v/v/v), vortexed at 1000 rpm for 10 minutes and transferred onto a deactivated glass vial insert (Agilent Technologies, ref: 5181–8872).

### Liquid Chromatography–Multiple Reaction Monitoring

Analyses were performed on a 1290 HPLC system (Agilent technologies) coupled to a 6490 triple quadrupole mass spectrometer with an Electro Spray Ionisation (ESI) source operating in the positive mode and in the MRM mode (Agilent technologies, Waldbronn, Germany). Data acquisition and analysis were performed using MassHunter workstation data acquisition and Masshunter Quantitative Analysis (version B07), respectively. Liquid chromatography (LC) separation was performed with a reversed-phase Zorbax 300 SB-C18 column kept at a temperature of 50 °C with a flow rate of 0.1 mL/min. A maximum pressure was set at 400 bars. The mobile phases consisted in (A) 0.1% FA in water containing 3% acetonitrile and (B) 0.1% formic acid in acetonitrile. The solvent gradient was set as follows (time, vol% solvent B): 0 min, 15; 0.2 min, 25; 4 min, 38; 4.5 min, 100; 5.5 min, 100; 6 min, 15 min. The ESI was set up using the following settings: capillary voltage 3000 V, gas flow 12 L/min at a temperature of 150 °C, sheath gas flow rate 7 L/min at a temperature of 250 °C, nebulizer at a pressure of 45 psi. Precursor ions were transferred into the first quadrupole with a cell accelerator voltage of 5 V, a high pressure ion funnel RF set at 150 V and a low pressure ion funnel RF set at 110 V. Collision energies were optimized for the peptide transitions of interest (Table 1). All transitions were used as qualifiers by automatic detection on specific retention time windows. One transition was used as quantifier.

### Calibration curves and quality control samples for orexin-A LC-MRM assays

Calibration curves were generate over a 0–200 pg/mL range (0, 4.0, 7.5, 10.0, 14.0, 19.0, 25.0, 50.0, 100.0, 200.0 pg/mL) in water/acetonitrile/formic acid (80/20/0.1, v-v:v) with unlabeled Orexin-A and spiked with SIS (50 pg/mL). Calibrator standards were vortexed for 10 minutes and transferred to deactivated glass vial inserts prior to (n = 5) LC-MRM analysis. The validity of the method was checked by carrying out linearity tests as defined in the EP17 guidelines published by the Clinical and Laboratory Standards Institute (CLSI)^38^, with 1/limits of blanks (LOB) defined by mean_blank+1.645 SD_blank), 2/limits of detection (LOD defined by mean_blank+3 (SD_blank)), and 3/limits of quantification (LOQ defined by mean_blank+10 (SD_blank)) of the area ratios between unlabeled Orexin-A/SIS standard)).

### Effects of dilution on integrity, stability, repeatability and accuracy

The effects of sample dilution were assessed by analyzing a CSF sample spiked with unlabeled light orexin-A standard (100 pg/mL) and diluted serially 3-, 6-, 9,- and 12-fold with a pool of blank CSF. A 500-μL aliquot of each dilution was taken and spiked with the SIS (50 pg/mL) before preparing the samples and performing LC-MRM measurements. The short-term stability of the analyte was assessed by measuring the calibration standards after storing them in the LC auto-sampler for several hours at 4 °C. The peak area ratios obtained (analyte/SIS) on orexin-A were analyzed to determine the stability of the analyte.

The repeatability of the LC-MS procedure was tested on all the calibration standards by measuring them five times in a row. The Relative Standard Deviation (RSD) and coefficient of variation (CV) were calculated from the area ratios obtained on unlabeled orexin-A/SIS. The repeatability was also determined in CSF samples. Pooled human CSF containing >200 pg/mL of orexin A quantified by RIA was measured five times and individual CSF samples (n = 10) with concentrations of orexin A ranging from 202 pg/mL to 391 pg/mL were measured in duplicate. To assess the reproducibility, calibration standards were prepared independently on three different days and measured five times each, and the inter-day precision was then calculated.

To assess the accuracy of the method, pooled blank CSF was spiked with 14, 25 and 50 mL of orexin A and all the samples were spiked with SIS (50 pg/mL). Sample preparation and MRM measurement were carried out as described in the case of CSF samples/above. The accuracy was calculated from the measured and theoretical concentrations, using the formula “Accuracy = [(calculated concentration)/(expected concentration)] ×100”.

The selectivity of the method was tested by comparing blank CSF samples and samples of CSF spiked with the orexin-A standard. MRM chromatograms of blank CSF samples were examined to detect any interferences in the retention time window of the orexin-A standard. Carry-over was determined by analyzing blank samples directly after LC-MRM runs with the highest calibration standards.

The occurrence of matrix effects was assessed by comparing the peak areas of the blank CSF spiked with SIS and spiked H_2_O/ACN/FA (80/20/0.1, v/v/v).

### Measurement of CSF orexin-A levels using RIA

Orexin-A quantification was performed in duplicate on CSF samples obtained from all the patients using I^125^ RIA kits from Phoenix Pharmaceuticals (Belmont, CA) in line with the manufacturer’s instructions. CSF Orexin-A levels below 110 pg/ml were defined as low, those ranging between 110 and 200 as intermediate, and those over 200 pg/ml as normal. All the values obtained were back-referenced to Stanford reference samples (Stanford University Center for Narcolepsy, Palo Alto CA).

### Statistical analysis

Percentages were used for categorical variables and medians and ranges for quantitative variables; the distribution of continuous variables was mostly skewed, according to the Shapiro-Wilk test. Mann-Whitney test were used to compare categorical variables between groups. Spearman’s rank correlation coefficient was used to compare two continuous variables. Significance level was set at p < 0.05. The Bland and Altman plot^39^ was also used to test the interchangeability of the methods. Statistical analyses were performed using Medcalc software program (15.2.2).

### Ethics Statement

The study protocol was approved by the local ethical committee (Comité de Protection des Personnes – Ile de France 06). The methods were carried out in accordance with the approved guidelines and regulations. Each participant signed legal consent forms. Informed consent was obtained from all subjects.

The samples/specimens were stored in a registered biological collection (#DC-2008-417) at the Montpellier CHU’s certified NFS 96-900 biobank (Ref: BB-0033-00031 www.biobanques.eu).

## Additional Information

**How to cite this article**: Hirtz, C. *et al*. From radioimmunoassay to mass spectrometry: a new method to quantify orexin-A (hypocretin-1) in cerebrospinal fluid. *Sci. Rep*. **6**, 25162; doi: 10.1038/srep25162 (2016).

## Figures and Tables

**Table 1:** MRM transitions for orexin-A with collision energy and resolution.

m/z precursor ion	Charge	MS1 Resolution	m/z product ion	Charge	Fragment	MS2 Resolution	Collision Energy (eV)
891.2 (901.4)	4+	wide	854.1 (862.4)	4+	b_32_-H_2_O	Wide	22
713.3 (721.1)	5+	wide	858.6 (866.9)	4+	b_29_	Wide	5
713.3 (721.1)	5+	wide	854.1 (862.4)	4+	b_32_-H_2_O	Wide	12
713.3 (721.1)	5+	wide	683.9 (690.2)	5+	b_32_-H_2_O	Wide	8

Corresponding data on the SIS are given in brackets. The transition used as the orexin-A quantifier is presented in bold print.

**Table 2:** Summary of LC-MS/MS conditions.

Column	Zorbax 300 SB-C18 (3.5 um, 1.0 × 150 mm i.d.)
Mobile phase A	0.1% Formic Acid/3% acetonitrile/96.9% water
Mobile phase B	0.1% Formic Acid/99.9% acetonitrile
	25–38% B for 3.8 min
Linear gradient	100% B for 1 min
	0% B for 4 min
Flow rate	100 μL/min
Injection Volume	20 μL in the full loop mode
Column temperature	50 °C
Polarity	Positive ESI
Capillary voltage	3000 Volts
Nebulizer	45 psi
Gas flow	12 L/min
Gas flow temperature	150 °C
Sheath gas flow rate	7 L/min
Sheath gas temperature	250 °C
High pressure ion funnel RF	150 V
Low pressure ion funnel RF	110 V
Cell Accelerator Voltage	6 V
Collision-induced dissociation gas	Nitrogen

**Table 3:** Method used to test the validity of the LC-MS orexin-A assay.

Calibration curve	0–200 pg/mL
Linearity	y = 0.0117x + 0.0001
R^2^	0.9885
LOB	0.006 pg/mL
LOD	0.009 pg/mL
LOQ	7.5 pg/mL
Analytical Precision	3–14%
Intra-assay CV	8%
Inter-assay	15%
Accuracy	94–104%
Carry-over	0%
Matrix effects	97%

**Table 4:** Accuracy of the LC-MS orexin-A assay.

Accuracy	
Orexin-A (pg/mL)	%
14	95
25	104
50	94

**Table 5:** Demographic, clinical and neurophysiological parameters of patients with narcolepsy type 1 (NT1) and control subjects.

	NT1 (n = 22)		Controls (n = 22)	
	n	%	n	%
Men	14	63.6%	6	27.3%
Women	8	36.4%	16	72.7%
Age at time of sampling, *in years*^(1)^	24.4 [7.1–62.6]		40.9 [3.3–67.4]	
BMI, *in kg/m*^*2*^ ^(1)^	23.3 [15.0–31.6]		23.7 [16.6–31.0]	
Age at onset, *in years*^(1)^	14.0 [5.0–48.0]			
Score at the Epworth Scale^(1)^	18 [11–24]			
Cataplexy	22	100.0%		
Hallucination^(2)^	9	50.0%		
Sleep Paralysis^(2)^	7	38.9%		
MSLT Latency, *in minutes*^(1)^	4.58 [0.20–8.00]			
Number of SOREMPS^(1)^	3 [2–5]			

^(1)^Continuous variables were expressed in median [minimum value-maximum value], ^(2)^Information available only in 18 NT1 patients.

## References

[b1] Dauvilliers Y., Arnulf I. & Mignot E. Narcolepsy with cataplexy. Lancet 369, 499–511, doi: 10.1016/S0140-6736(07)60237-2 (2007). 17292770

[b2] Mieda M. & Sakurai T. Overview of orexin/hypocretin system. Progress in brain research 198, 5–14, doi: 10.1016/B978-0-444-59489-1.00002-1 (2012). 22813966

[b3] Sakurai T. . Orexins and orexin receptors: a family of hypothalamic neuropeptides and G protein-coupled receptors that regulate feeding behavior. Cell 92, 1 page following 696 (1998). 10.1016/s0092-8674(02)09256-5 9527442

[b4] Peyron C. . Neurons containing hypocretin (orexin) project to multiple neuronal systems. The Journal of neuroscience the official journal of the Society for Neuroscience 18, 9996–10015 (1998). 9822755 10.1523/JNEUROSCI.18-23-09996.1998 PMC6793310

[b5] Horvath T. L. . Hypocretin (orexin) activation and synaptic innervation of the locus coeruleus noradrenergic system. The Journal of comparative neurology 415, 145–159 (1999). 10545156

[b6] Kilduff T. S. & Peyron C. The hypocretin/orexin ligand-receptor system: implications for sleep and sleep disorders. Trends in neurosciences 23, 359–365 (2000). 10906799 10.1016/s0166-2236(00)01594-0

[b7] Shan L., Dauvilliers Y. & Siegel J. M. Interactions of the histamine and hypocretin systems in CNS disorders. Nature reviews. Neurology 11, 401–413, doi: 10.1038/nrneurol.2015.99 (2015). 26100750 PMC8744538

[b8] Chemelli R. M. . Narcolepsy in orexin knockout mice: molecular genetics of sleep regulation. Cell 98, 437–451 (1999). 10481909 10.1016/s0092-8674(00)81973-x

[b9] Taheri S., Zeitzer J. M. & Mignot E. The role of hypocretins (orexins) in sleep regulation and narcolepsy. Annual review of neuroscience 25, 283–313, doi: 10.1146/annurev.neuro.25.112701.142826 (2002). 12052911

[b10] Thannickal T. C. . Reduced number of hypocretin neurons in human narcolepsy. Neuron 27, 469–474 (2000). 11055430 10.1016/s0896-6273(00)00058-1 PMC8760623

[b11] Peyron C. . A mutation in a case of early onset narcolepsy and a generalized absence of hypocretin peptides in human narcoleptic brains. Nature medicine 6, 991–997, doi: 10.1038/79690 (2000). 10973318

[b12] Thorpy M. J. In American Academy of Sleep Medicine. International Classification of Sleep Disorders 3rd ed (eds ICSD) Westchester, IL, Ch. Diagnostic Criteria, 21–24 (2014).

[b13] Dauvilliers Y. A., Lehmann S., Jaussent I. & Gabelle A. Hypocretin and brain beta-amyloid peptide interactions in cognitive disorders and narcolepsy. Frontiers in aging neuroscience 6, 119, doi: 10.3389/fnagi.2014.00119 (2014). 24966833 PMC4052448

[b14] Liguori C. . Orexinergic system dysregulation, sleep impairment, and cognitive decline in Alzheimer disease. JAMA neurology 71, 1498–1505, doi: 10.1001/jamaneurol.2014.2510 (2014). 25322206

[b15] Kang J. E. . Amyloid-beta dynamics are regulated by orexin and the sleep-wake cycle. Science 326, 1005–1007, doi: 10.1126/science.1180962 (2009). 19779148 PMC2789838

[b16] Mignot E. . The role of cerebrospinal fluid hypocretin measurement in the diagnosis of narcolepsy and other hypersomnias. Archives of neurology 59, 1553–1562 (2002). 12374492 10.1001/archneur.59.10.1553

[b17] Liguori C. . CSF beta-amyloid levels are altered in narcolepsy: a link with the inflammatory hypothesis? Journal of sleep research 23, 420–424, doi: 10.1111/jsr.12130 (2014). 24635662

[b18] Schmidt F. M. . Cerebrospinal fluid melanin-concentrating hormone (MCH) and hypocretin-1 (HCRT-1, orexin-A) in Alzheimer’s disease. PloS one 8, e63136, doi: 10.1371/journal.pone.0063136 (2013). 23667582 PMC3646736

[b19] Percy A. J., Chambers A. G., Yang J., Hardie D. B. & Borchers C. H. Advances in multiplexed MRM-based protein biomarker quantitation toward clinical utility. Biochimica et biophysica acta 1844, 917–926, doi: 10.1016/j.bbapap.2013.06.008 (2014). 23806606

[b20] Lehmann S. . Quantitative Clinical Chemistry Proteomics (qCCP) using mass spectrometry: general characteristics and application. Clinical chemistry and laboratory medicine : CCLM / FESCC 51, 919–935, doi: 10.1515/cclm-2012-0723 (2013). 23183755

[b21] Addona T. A. . Multi-site assessment of the precision and reproducibility of multiple reaction monitoring-based measurements of proteins in plasma. Nature biotechnology 27, 633–641, doi: 10.1038/nbt.1546 (2009). PMC2855883 19561596

[b22] Picotti P. & Aebersold R. Selected reaction monitoring-based proteomics: workflows, potential, pitfalls and future directions. Nature methods 9, 555–566, doi: 10.1038/nmeth.2015 (2012). 22669653

[b23] Lehmann S. . Comparison of Hydrophobic, Lipophilic and Immunodepletion PreFractionation Methods for Label-Free LC-MS/MS Identification of Biomarkers in Human Cerebrospinal Fluid. Proteomics & Bioinformatics S5 003, doi: 010.4172/jpb.S4175-4003 (2014).

[b24] Yin J., Mobarec J. C., Kolb P. & Rosenbaum D. M. Crystal structure of the human OX2 orexin receptor bound to the insomnia drug suvorexant. Nature 519, 247–250, doi: 10.1038/nature14035 (2015). 25533960

[b25] Roh J. H. . Potential role of orexin and sleep modulation in the pathogenesis of Alzheimer’s disease. The Journal of experimental medicine 211, 2487–2496, doi: 10.1084/jem.20141788 (2014). 25422493 PMC4267230

[b26] Lucey B. P. & Bateman R. J. Amyloid-beta diurnal pattern: possible role of sleep in Alzheimer’s disease pathogenesis. Neurobiology of aging 35 Suppl 2, S29–34, doi: 10.1016/j.neurobiolaging.2014.03.035 (2014). 24910393

[b27] Lucey B. P. & Holtzman D. M. How amyloid, sleep and memory connect. Nature neuroscience 18, 933–934, doi: 10.1038/nn.4048 (2015). 26108720 PMC4770804

[b28] Kroll M. H. & Elin R. J. Interference with clinical laboratory analyses. Clinical chemistry 40, 1996–2005 (1994). 7955368

[b29] Weber T. H., Kapyaho K. I. & Tanner P. Endogenous interference in immunoassays in clinical chemistry. A review. Scandinavian journal of clinical and laboratory investigation. Supplementum 201, 77–82 (1990). 2244186

[b30] Marks V. False-positive immunoassay results: a multicenter survey of erroneous immunoassay results from assays of 74 analytes in 10 donors from 66 laboratories in seven countries. Clinical chemistry 48, 2008–2016 (2002). 12406987

[b31] Lehmann S. . Quantitative Clinical Chemistry Proteomics (qCCP) using mass spectrometry: general characteristics and application. Clinical Chemistry and Laboratory Medicine 51, 919–935 (2013). 23183755 10.1515/cclm-2012-0723

[b32] Hortin G. L. & Sviridov D. The dynamic range problem in the analysis of the plasma proteome. Journal of proteomics 73, 629–636, doi: 10.1016/j.jprot.2009.07.001 (2010). 19619681

[b33] Dauvilliers Y., Siegel J. M., Lopez R., Torontali Z. A. & Peever J. H. Cataplexy--clinical aspects, pathophysiology and management strategy. Nature reviews. Neurology 10, 386–395, doi: 10.1038/nrneurol.2014.97 (2014). PMC8788644 24890646

[b34] Gerashchenko D. . Relationship between CSF hypocretin levels and hypocretin neuronal loss. Experimental neurology 184, 1010–1016, doi: 10.1016/S0014-4886(03)00388-1 (2003). 14769395

[b35] Dauvilliers Y. . CSF hypocretin-1 levels in narcolepsy, Kleine-Levin syndrome, and other hypersomnias and neurological conditions. Journal of neurology, neurosurgery, and psychiatry 74, 1667–1673 (2003). 10.1136/jnnp.74.12.1667 PMC1757412 14638887

[b36] Thannickal T. C., Nienhuis R. & Siegel J. M. Localized loss of hypocretin (orexin) cells in narcolepsy without cataplexy. Sleep 32, 993–998 (2009). 19725250 10.1093/sleep/32.8.993 PMC2717206

[b37] Fronczek R. . Hypocretin (orexin) loss in Alzheimer’s disease. Neurobiology of aging 33, 1642–1650, doi: 10.1016/j.neurobiolaging.2011.03.014 (2012). 21546124

[b38] Moretti M., Sisti D., Rocchi M. B. & Delprete E. CLSI EP17-A protocol: a useful tool for better understanding the low end performance of total prostate-specific antigen assays. Clinica chimica acta; international journal of clinical chemistry 412, 1143–1145, doi: 10.1016/j.cca.2011.03.002 (2011). 21396930

[b39] Bland J. M. & Altman D. G. Statistical methods for assessing agreement between two methods of clinical measurement. Lancet 1, 307–310 (1986). 2868172

